# Using Kern model to design, implement, and evaluate an infection control program for improving knowledge and performance among undergraduate nursing students: a mixed methods study

**DOI:** 10.1186/s12909-023-04775-3

**Published:** 2023-10-25

**Authors:** Haydeh Heidari, Zahra Hossein mirzaee Beni, Fatemeh Deris

**Affiliations:** 1 Modeling in Health Research Center, Faculty of Nursing and Midwifery, Shahrekord University of Medical Sciences, Shahrekord, Iran; 2 School of Nursing and Midwifery, Shahrekord University of Medical Sciences, Rahmatieh, PO Box 3833346699, Shahrekord, Iran; 3 Shahrekord University of Medical Sciences, Shahrekord, Iran

**Keywords:** Nursing student, Infection, Mixed method, Kern

## Abstract

**Background:**

Nurses and Nursing students are the front line of care in managing the care of infectious patients then they have more contact with patients than other students. Therefore, the aim of this study was designing, implementing and evaluating the infection control program among undergraduate nursing students using a mixed study.

**Method:**

The mixed method approach with sequential exploratory (qualitative-quantitative) method was used. Kern model was applied in six steps included: 1-Needs assessment 2- Initial design 3- Goals and specific objectives 4- Educational strategy 5- Program implementation 6- Program evaluation. Thirty nursing students and 3 nursing faculty members were selected through purposive sampling for focus group in need assessment. Single-group semi-experimental study with a pre-test and post-test design was used in partnership with all eighth semester nursing students in program evaluation.

**Result:**

Data analysis of focus group obtained two categories including: Need to improve knowledge in infection control and need to improve performance in infection control. With need assessment and literature review, educational content developed according to nursing students learning needs. Then, 3 faculty members prepared a course plan including goals, specific objectives, educational strategy for student assignments**.** One-way analysis of variance to compare the average score of knowledge, performance and its categories before, 2 weeks after the intervention and 2 months after the intervention shows a statistically significant difference (*p >* 0.001). Pearson's correlation coefficient shows that there is a negative linear relationship between work experience and knowledge score 2 weeks after and 2 months after the intervention (*p <* 0.05).

**Conclusion:**

According to our results, it is recommended the need to revise the curriculum for the integration of the infection control program in the undergraduate nursing education**.** Of course, it is necessary to conduct more studies in the field by dividing this program into internship and field internship.

## Background

One of the challenges in the field of healthcare is the increase in the occurrence of highly transmissible and contagious diseases in lower-middle income families in recent decades [1]. In total, more than half of the fifty million deaths that occur in the world are directly related to infectious diseases [2]. Infectious diseases are a major problem all over the world, and due to the significant impact these diseases have had on global health, economy, and social stability of human society, it is necessary for the health system to design and implement new interventions and care [3].

Undoubtedly, the most effective, least expensive and most desirable method of managing infection at any time and place is to prevent its occurrence [4]. Any action that can help to control and prevent the spread of infection is called infection control, which requires a team approach and each team member must apply infection control methods correctly [5]. For this purpose, the World Health Organization has designed evidence-based infection prevention and control guidelines in order to improve the quality of care services and reduce mortality [6].

Nurses are considered the most important workers in infection control and prevention, accounting for the majority of the global health workforce and the largest health care expenditure [7–9]. Nursing students have a great responsibility to protect themselves, their families and their patients from infection [10]. Various studies in low- and middle- income countries showed that nursing students do not have sufficient knowledge and practice in the field of infection control [11, 12]. Nurses and nursing students are the front line of care in managing the care of infectious patients [10].

Adequate knowledge of infection control is essential to assist new nursing graduates to work as novice level health care providers in health care settings, to reduce infection risks and provide safe patient care by minimizing patient-to-patient infection [13]. However, one study has reported that, there is poor knowledge of students regarding infection control and prevention practices in undergraduate nursing programs [14]. Researcher reported that due to the nature of care, nursing students have more contact with patients than other students, according to these reports, more than half of nursing students had a history of needle sticks and more than a third of them had a history of contact with blood and secretions [15]. Another researcher stated that infection control programs should be added to the undergraduate curriculum [16]. Another researcher stated that infection control education for nursing students should be based on reliable sources and continuously updated [17].

The goal of clinical education is to prepare nursing students to accept different roles in the health system; one of the challenges of nursing schools around the world is training students who can provide optimal care in complex clinical conditions [18, 19]. Extensive changes in clinical environments have determined the need for changes in clinical educational planning. It is the duty of the clinical trainers to be constantly informed about these developments and to design evidence-based programs as much as possible, so as to provide better quality clinical training to the students [20]. The results of the systematic review showed that although it is necessary to teach nursing students about infection control, there is no standard approach for teaching this to students [21].

The general purpose of providing community health nursing courses is to transfer knowledge and necessary information to students in the field of community health, family concepts and environmental health issues, so based on this, students can apply the principles of health services in the form of nursing process, and provide appropriate measures to solve the problems of the society and health problems of the family [22].

Considering that the knowledge of nursing students about infection control is low [14, 15] and since students have the responsibility of taking care of themselves, their families and patients, and on the other hand, nursing education teachers have the responsibility of revising the curriculum based on evidence, we decided to conduct a study with the aim of designing, implementing and evaluating the infection control program among undergraduate nursing students using a mixed study.

### Research objectives

✓ To determine the needs assessment of the infection control program
✓ To determine the educational content of the infection control program
✓ To determine educational goals and teaching methods
✓ To study the effect of infection control program for improving knowledge and performance among undergraduate nursing students

## Methods

### Study design and settings

The mixed method approach with sequential exploratory (qualitative-quantitative) method was used [23]. The study setting was Shahrekord School of Nursing and Midwifery affiliated to Shahrekord University of Medical Sciences in Iran. Kern model was applied in six stages (six steps) to develop infection control program in nursing students that included:

1. Needs assessment
2. Initial design
3. Goals and specific objectives
4. Educational strategy
5. Program implementation
6. Program evaluation [24]

### Step 1: needs assessment

In the first and second stages, this was done by assessing the needs of the students through focus groups and conducting interviews. The group members were purposefully selected from people who had the required information and were willing to participate in the study. Several factors should be considered in choosing the number of focus group members. Some studies suggested the number of focus group members between 4 and 15 participants [25, 26]. However, the researcher selected purposely thirty nursing students and 3 nursing faculty members according to the maximum diversity in participants, subject, education level and gender in this study. Inclusion criteria were nursing students, who had enrolled in internship nursing and who were interested to share their experiences, nursing faculty members who had Bachelor’s and higher degrees in nursing with infection control program credit. The exclusion criterion was unwillingness to participate in the study. The first author, who was assistant professor and hold infection control credit, conducted the interviews with eligible participants. Management of focus group sessions was in the form of group discussion in nursing students. Attempts were made to use the experiences of various students at this stage. First, it should be noted that the consent of the students to participate in the meetings was obtained. The focus group interview started with an open question and continued with probing questions (Table 1). Interviews were conducted with 30 students in 3 focus sessions and 3 nursing faculty members with 1 focus session in rest room in hospital. The average duration of the interview was 45 minutes; the interviews were analyzed in the form of conventional qualitative content analysis. First, the recorded interviews were transcribed. The texts was reviewed several times. Then, meaning units were extracted from the participants' statements in the form of primary codes. Codes were also classified based on semantic and conceptual similarity in the subcategories. Finally, the data were classified in the main categories that are more general and conceptual [27].

**Table 1:** Descriptive indices of demographic variables in the studied samples (*n=*56)

Variables		*N* (%)
Gender	Female	27 (48.2)
	Male	29 (51.8)
Employment history in health care centers	Yes	1 (1.8)
	No	55 (98.2)
Employment history in other organizations	Yes	1 (1.8)
	No	55 (98.2)
Work shift	Morning	46 (90.2)
	Evening	5 (9.8)
History of participating in the previous infection control workshop	Yes	43 (78.2)
	No	12 (21.8)
history of needle stick injury	Yes	21 (37.5)
	No	35 (62.5)
work experience (years) (Range of values)Mean± SD	(0-8) 0.18 ± 1.10	
Workshop duration (hours) (Range of values) Mean± SD	(0-8) 3.40 ± 2.10	
The number of needle sticks (Range of values) Mean± SD	(0-3) 0.53 ± 0.87	

For assessing trustworthiness, four criteria: credibility, dependability, confirmability, and transferability were used [28].

### Step 2: initial design

Initial design infection program developed an initial design infection program according qualitative phase and literature review [5, 29–32]. First, the initial draft of the infection control program was prepared based on the qualitative findings of the phased needs assessment. Then the educational content and approaches were determined based on the literature review. Of course, it should be noted that the questionnaires available in Iran were extracted for the evaluation step by literature review.

### Step 3: goals and specific objectives

Specific cognitive and skill objectives of the infection control program were designed based on concepts such as infection control with emphasis on the concepts of infection, hospital infections, prevention of infection transmission, use of personal protective equipment, principles of hygienic hospital environment, isolation, infection control of general and special unit such as dialysis, operating room, NICU.

### Step 4: educational strategy

In this step, group discussion, question and answer, scenario presentation and role playing were used to teach the content of the infection control program.

### Steps 5: implementation and monitoring of the infection control program (quasi ‑ experimental)

Single-group semi-experimental study with a pre-test and post-test design was used, which was conducted with the aim of determining the effect of integrating the infection control training program into the nursing undergraduate curriculum on the knowledge and practice of infection control of nursing students. The research population consisted of all eighth semester nursing students of the Faculty of Nursing and Midwifery, Shahrekord University of Medical Sciences, who were selected by census method. This group of students has been selected because they passed all the necessary prerequisites to attend the relevant general clinical units, operating room and ICU, neonatal and NICU, dialysis department. The criteria for entering the study included studying nursing at the Faculty of Nursing and Midwifery of Shahrekord University of Medical Sciences, being in the eighth semester, taking a course, and being willing to participate in the study. Exclusion criteria included not wanting to continue participating in the research, removing the course unit, not returning the relevant questionnaires, and not participating in at least one class session.

The infection control educational program was prepared based on Kern's stages. This program was trained by the educational instructor during three days of 6-hours in the field internship and using a strategy which included lectures, group discussions, role playing, scenario and questions and answers. The data before and after the intervention were compared according to the objectives of the research. During the field internship period, the student's performance was reviewed and feedback was given to them.

The outcome investigated in this study was the knowledge and practice of infection control of nursing students. Data collection was self-reported and using two-part questionnaires that included demographic characteristics, knowledge and infection control performance questionnaires, which were conducted at the beginning of the study, 2 weeks later, and 8 weeks after the end of the intervention. Demographic characteristics questionnaire included personal characteristics of gender, age, educational qualification, employment status in health care centers, employment status in other organizations, years of work experience, history of participation in infection control workshops and the amount of benefit from weekly conferences related to hospital infection control, work shift, a history of needle stick, and the number of times of needle stick.

The first version of the infection control knowledge and practice questionnaire was designed and psychometrically tested by Saberi et al. (2012) [32]. To adapt the questionnaire to measure the infection control variable, the researchers made changes in the items of the questionnaire with the permission of the main author and by reviewing the texts, and based on the needs assessment of the first stage (focus group). Academic semester items, employment history in health care centers, employment history in other organizations, and history of needle stick were added to the demographic profile section. Nine questions were added to the performance section that included 6 questions related to compliance with standard precautions, 2 questions related to isolation and 1 question related to wound care section. In the knowledge section, 5 questions related to the concepts of infection, occupational exposure, infection control in neonatal unit and CSR were added. The final questionnaire is arranged in three parts, the first part is related to the personal characteristics of the samples, including gender, age, level of education, employment status, work history, history of participation in the hospital infection control workshop and the amount of participation in weekly conferences related to hospital infection control (per hour).The second part contains 14 questions related to self-reporting of subjects' performance in the fields of hand hygiene (4 questions), wound care (4 questions), urinary infection prevention (2 questions), respiratory infection prevention (2 questions), and vein catheter care (2 questions). The performance of the samples was assessed regarding each of the mentioned behaviors and is based on a 5-point Likert scale: 0- I don't do it at all, 1- I rarely do it, 2- I do it sometimes, 3-I do it most of the time 4-I always do it.

In the third part of the questionnaire, with 10 four-choice questions related to the process of infection, the factors involved in the occurrence and prevention of hospital infection, the role of nurses in hospital infection control, and the level of knowledge of the subjects in this regard will be measured. The number of correct answers indicated the level of awareness, the range of the awareness score was considered to be 0-10. Questions of the knowledge section were in the form of yes, no, and I do not know. Score of 1 was given to the correct answers and the wrong answers and “I do not know” received the score of zero [32]. The scientific validity of the questionnaire was determined by the content validity method, scientific reliability for knowledge section was determined by the retest method, and the correlation coefficient was 89% for the knowledge. Cronbach’s alpha was applied to obtain the reliability of the performance section and the alpha value was calculated to be 0.86. Factor analysis was not done.

### Data analysis

In this study, descriptive statistics (frequency distribution tables - calculation of numerical indices) and inferential statistics (one-way analysis of variance, Tukey's post hoc test, Pearson's correlation coefficient, and independent t test) were used to analyze the data. The value of *p<*0.05 was considered as the significant level. SPSS 21 software was used to analyze the data.

## Result

### Demographic information

Fifty-six people participated in this study. The majority of the samples was female, had no employment history in health care centers, morning shift and had a history of participation in the infection control workshop (Table 1). 48.2% of the participants were female and 58.2% were male. 1.8% of them had experience working in health centers. 1.8% of them had work experience in other organizations. 90.2% worked the morning shift and 9.8% worked the evening shift. 78.2% had a history of participating in the previous infection control workshop. 37.5% of the participants had a history of needle stick.

### Main result

Six steps of Kern model were used for designing, implementing and evaluating infection control program.

### Step 1, 2, 3, 4

Data analysis of focus group obtained two categories including: Need to improve knowledge in infection control and Need to improve performance in infection control (Table 2). With need assessment and literature review, educational content developed according to nursing students learning needs. Then, 3 faculty members prepared a course plan including goals, specific objectives, educational strategy for student assignments (Table 3).

**Table 2:** Qualitative focus group

**Categories**	**Subcategories**	**Quotations**
**Need to improve knowledge in infection control**	**Necessity of learning in the field of needle stick** **The necessity of learning the main concepts of infection control** **Necessity of learning in the field of infection control in general unit** **The necessity of learning infection control in intensive unit**	*If we get a needle stick, we should not press the place because the blood flow will increase in that direction if we press it, and the contamination will spread. Just wash it with clean soap and water [FG1].* *If we get a needle stick, we have to take medicine, I don't remember the name. We should take medicine if the person is positive. Even if it is negative, we take medicine because it may be a false negative[FG1]* *The trainers told us a series of general principles first, for example, the operating room, I don't know the other departments, but, for example, the first thing that we have to follow in the departments is hand washing [FG2]* *Nails should be kept short in the neonatal intensive care unit. Most of the children have sepsis. The infection control rule was that if you touch an infant, you must wash your hands. Their incubator should be cleaned, but we don't know how many days and how….[FG1]* *As a teacher with several years of experience working with students, I think that due to the importance of infection control, it is necessary that the infection control program should take into account the topics of increasing knowledge and improving practical work [fm2]*
**Need to improve performance in infection control**	**The necessity of performing the correct principles of suction** **The necessity of performing the isolation process** **The need to do infection control actions in general and specialized units**	*Dressing or procedures that need to be sterile, such as bladder catheterization and suction,* I don’t know if we are doing these correct, that is, how should we do it correctly?* [FG2].* *We should have an isolation room. I don't know if the isolation room is only used for respiratory patients…[FG1].* *Many times we go to the infection control workshop, but we don't learn practical work* *I mean, now that we have learned the information, let's go to the general and specialized units (such as NICU, ICU,..) to see the infection control standards of the departments and do practical work [FG2].* *Regarding the improvement of the practical work of students in specialized departments such as dialysis and NCU, it is necessary to implement the program in the field internship period where the student has passed all the necessary prerequisites[fm1]*

**Table 3:** Course plan on infection control

**Goals: To improve knowledge and practice of infection control among undergraduate nursing students**			
**Cognitive objectives**	**Educational strategy**	**Student assignments**	**Evaluation**
1. Explain the concepts of infectious diseases, and hospital infections	Group discussion, question and answer, scenario, presentation Role playing	Book reading about infection control Investigation of infection control in clinical setting Assessing infection control problems in health setting Prioritizing problems Developing short term goals Providing nursing process	Assessing assignments & feedback Questionnaire pretest & post test
2. Explain the importance and types of hospital infections.			
3. Describe the duties of infection control committees.			
4. Explain how to control infection in general departments.			
5. Describe how to control infection in the operating room & ICU.			
6. Describe how to control infection in the C.S.R. unit.			
7. Explain how to control infection in the NICU			
8. Explain how to control infection in the dialysis department			
9. Describe standard precautions.			
10. Discuss the observance of the correct principles of hand hygiene and safety protective equipment (PPE).			
11. Explain the principles of isolation.			
12. Explain occupational exposure management & needle stick.			
13. Explain about safe injection techniques.			
14. Explain the principles of observing sterilization in performing aseptic procedures			
**Skill objectives (psychomotor)**			
1. Perform hand washing and hand rub methods correctly.			
2. Observe the order of using personal protective equipment correctly.			
3. Wear and remove personal protective equipment correctly.			
4. Open and removes sterile instruments and sets correctly.			
5. Perform intramuscular, subcutaneous and intravenous injections in compliance with aseptic principles.			
6. Separate the waste properly.			
7. Correctly use disinfectants and materials.			
8. Observe aseptic principles in using tools & equipment.			
9. Suction the respiratory secretions correctly			

### Step 5, 6 implementation and monitoring of the infection control program (quasi ‑ experimental)

One-way analysis of variance to compare the average score of knowledge, performance and its categories before, 2 weeks after the intervention and 2 months after the intervention shows a statistically significant difference (*p >* 0.001). Tukey's post-hoc test shows that the average score of knowledge, performance and its domains from the time before the intervention is significantly lower than the times 2 weeks after and 2 months after the intervention (*p <* 0.001). However, no significant difference was observed between the average score of knowledge, performance and its areas between 2 weeks and 2 months after the intervention (*p<*0.05) (Table 4).

**Table 4:** Determining the values of descriptive indicators for Knowledge, Practice and Practice domains in the studied samples during the investigated times (*n=*56)

Variables	Time of intervention	Mean± SD	Range of values	*P*- value
Total score Knowledge	Before	7.48 ± 1.76	(4-12)	< 0.001^*^
	2 weeks after the intervention	10.82 ± 1.97	(5-14)	
	2 months after the intervention	11.16 ± 2.07	(5-15)	
Hand hygiene	Before	18.30 ± 2.66	(11-23)	< 0.001*
	2 weeks after the intervention	21.73 ± 2.93	(15-27)	
	2 months after the intervention	21.89 ± 2.87	(16-27)	
Wound caring	Before	15.52 ± 3.19	(7-20)	< 0.001*
	2 weeks after the intervention	17.27 ± 2.16	(11-20)	
	2 months after the intervention	17.39 ± 2.14	(11-20)	
Prevention of respiratory infection	Before	9.34 ± 2.19	(2-13)	< 0.001*
	2 weeks after the intervention	12.14 ± 2.53	(4-16)	
	2 months after the intervention	12.25 ± 2.47	(4-16)	
Venous catheter care	Before	5.07 ± 1.61	(0-8)	< 0.001*
	2 weeks after the intervention	6.46 ± 1.14	(3-8)	
	2 months after the intervention	6.50 ± 1.14	(3-8)	
Prevention of urinary tract infection	Before	5.52 ± 1.61	(1-8)	< 0.001*
	2 weeks after the intervention	6.55 ± 1.25	(3-8)	
	2 months after the intervention	6.55 ± 1.25	(3-8)	
Prevention of contact infection	Before	5.64 ± 1.57	(2-9)	< 0.001*
	2 weeks after the intervention	8.52 ± 1.88	(5-12)	
	2 months after the intervention	8.50 ± 1.92	(4-12)	
Total score of practice	Before	59.39 ± 8.13	(31-73)	< 0.001*
	2 weeks after the intervention	72.68 ± 8.72	(45-88)	
	2 months after the intervention	73.09 ± 8.48	(46-88)	

^*^The results of the One-way ANOVA and Tukey post hoc test

Pearson's correlation coefficient shows that there is a negative linear relationship between work experience and knowledge score 2 weeks after and 2 months after the intervention (*p <* 0.05). With the increase of work experience, the knowledge score decreased 2 weeks after and two months after the intervention. Also, the Pearson correlation coefficient between work experience and performance score shows a negative linear relationship before, 2 weeks after and 2 months after the intervention (*p<*0.01). With increasing work experience, the performance score before, 2 weeks after and 2 months after the intervention decreased. However, no significant linear relationship was observed between the variables of workshop hours and needle frequency with knowledge and performance scores (*p<*0.05) (Table 5).

**Table 5:** Pearson's correlation for the relationship between work experience, hours of participation in previous infection control workshops and the number of needle sticks with the knowledge and practice scores during the investigated times (*n=*56)

Variables	Time of intervention	work experience (years)	Workshop duration (hours)	The number of needle sticks
Knowledge	Before	*r=*- 0.008 *p=* 0.995	*r=* 0.031 *p=* 0.823	*r=* - 0.003 *p=* 0.981
	2 weeks after the intervention	*r=*- 0.422 *p=* 0.001^a^	*r=*- 0.080 *p=* 0.562	*r=*- 0.012 *p=* 0.932
	2 months after the intervention	*r=* - 0.301 *p=* 0.024^a^	*r=* 0.006 *p=* 0.966	*r=* 0.033 *p=* 0.815
Practice	Before	*r=* - 0.510 *p<* 0.001^a^	*r=* - 0.005 *p=* 0.970	*r=* - 0.089 *p=* 0.524
	2weeks after the intervention	*r=* - 0.412 *p=* 0.001^a^	*r=* - 0.128 *p=* 0.352	*r=* - 0.031 *p=* 0.825
	2 months after the intervention	*r=* - 0.412 *p=* 0.002^a^	*r=* 0.109 *p=* 0.430	*r=* - 0.025 *p=* 0.861

^a^The results of the Pearson's correlation

The independent t-test to compare the knowledge score and performance shows that in each of the previous times, 2 weeks after and 2 months after the intervention for qualitative demographic variables, only the average knowledge score of female students was significantly higher than the knowledge score of male students (*p<*0.05). But in other variables, no significant difference was observed in knowledge and performance scores (*p<*0.05) (Table 6).

**Table 6:** Comparison average score of knowledge and practice with qualitative demographic variables in the studied samples (*n=*56)

		Before	2 weeks after the intervention	2 months after the intervention	Before	2 weeks after the intervention	2 months after the intervention
Gender	Female	7.62±1.88	11.34±1.72	11.66±1.59	60.69±6.76	72.76±8.59	73.10±8.35
	Male	7.33±1.64	10.26±2.10	10.63±2.40	58.00±9.31	72.59±9.02	73.07±8.77
*P*- value		0.546	0.039^a^	0.063	0.219	0.944	0.990
History of participating in the previous infection control workshop	Yes	7.75±1.87	10.72±2.10	11.12±2.23	59.07±8.40	71.72±9.10	72.23±8.87
	No	7.42±1.44	11.17±1.59	11.33±1.61	61.00±7.40	75.08±6.35	75.17±6.24
*P*- value		0.871	0.498	0.753	0.474	0.237	0.289
history of needle stick injury	Yes	7.43±1.75	11.24±2.45	11.57±2.89	57.67±7.28	73.29±9.96	73.76±9.66
	No	7.51±1.79	10.72±1.61	10.91±1.36	60.43±8.53	72.31±8.02	72.69±7.81
*P*- value		0.862	0.224	0.254	0.221	0.690	0.650
Work shift	Morning	7.52±1.67	10.61±1.99	10.96±2.09	59.57±7.85	72.73±8.76	72.76±8.48
	Evening	7.20±2.95	11.20±1.30	11.60±1.52	55.20±5.54	74.20±7.98	75.00±7.42
*P*- value		0.707	0.522	0.507	0.234	0.657	0.574

^a^The results of the independent t-test

## Discussion

The results of a qualitative study showed that 51% of nursing students stated that they were weak about infection control in the clinical environment; they recommended that nursing educators should design an infection control curriculum using appropriate teaching methods for the clinical environment of students [33]. The present study was conducted with the aim of designing, implementing and evaluating the infection control program in nursing students. This study is the first study in Iran that designed, implemented and evaluated the infection control program in community health internship in nursing students. One of its features is the use of qualitative and quantitative methods in its development, implementation and evaluation. In the current study, we used needs assessment to develop an infection control plan, while a researcher stated that one of the essential components in developing a training program for educational teachers is needs assessment based on the organizational context in society [34]. In support of our findings, many studies used educational models in designing and implementing and evaluating educational programs and revising the nursing curriculum [35]. Educational models are a suitable approach for revising curriculum due to covering all the steps [36].

The results of the needs assessment and our qualitative part showed that students need an infection control educational program in the context of knowledge and performance, while the results of a qualitative researcher in Ghana in 2021 showed that students' knowledge about infection control is high, but their performance is weak [37]. An important feature in the study is the design of the infection control program in the final year of nursing education. One researcher confirmed that t students need ongoing education on infection prevention and infection control guidelines in the final year of the nursing program as well as the internship [38]. One of the characteristics of the infection control program in our study was the existence of a specific program in special units such as NICU, because nursing students do not know about infection control in such units due to insufficient experience and practical limitations in these units. Also, the results of a study showed that teaching the infection control program in NICU increases the efficiency and satisfaction of nursing students [39].

The results of this research show that the average score of knowledge, performance and its dimensions after the intervention increased significantly in 2 weeks after the intervention. Another study in Egypt concluded that the topics of infection prevention and control in nursing undergraduate courses are insufficient and need to be updated [38].

Considering the importance of infection control, it is emphasized that more focus should be placed on teaching the content of infection control during nursing education.

The findings of the present study indicated that the average score of knowledge, performance and its dimensions increased significantly 2 months after the intervention. The strength of the present study is the implementation of the infection control training program in the form of a comprehensive training program in the field during three days. A researcher in 2018 showed that the use of an online learning module improved knowledge in the field of infection control among undergraduate nursing students. They also suggested that the standard infection control program should be strengthened in all nursing schools [40].

Our findings show that only the mean knowledge score of female students was significantly higher than the knowledge score of male students after the intervention. It seems that female students have a greater desire to increase and improve the level of knowledge. Although in our study, female students had a higher score only in the dimension of knowledge, one research in 2021 confirmed our result [41]; but the results of a study in 2021 showed that female students had a higher score in both dimensions of knowledge and performance [42].

There are conflicting studies on the gender and knowledge and performance scores of nurses [41, 42]. We suggest education of infection control programs is essential for both genders.

The results of the present study show that there is a negative linear relationship between work experience and knowledge score and performance 2 weeks after and 2 months after the intervention. With the increase of work experience, the score of knowledge and performance decreased 2 weeks after and two months after the intervention. Other studies did not confirm our findings. The results of study showed that the more the work experience, the more the knowledge of infection control [41]. It seems that in the current study, sufficient and practical training for nurses is not provided to improve their knowledge and performance in the field of infection control.

Our study show no significant relationship was observed between the variables of hours of participation in previous infection control workshops and the number of times of needle stick with knowledge and performance scores. It seems that the previous infection control workshops held for nursing students were lectures and short-term, it is suggested that the infection control training program be implemented in a comprehensive theoretical and practical manner and in a longer period of time. It seems that people who have a higher work experience, especially in practice nurse, due to lack of updating nursing knowledge , lack of participation in regular and comprehensive training programs, decrease motivation to learn, have been reported as having lower knowledge and lower performance scores.

The findings of our study have implications for nursing professors, curriculum developers and clinical mangers. In order to prepare nursing expert students to enter the clinical field, it is necessary to add the infection control program to the undergraduate curriculum. Of course, it is necessary for the clinical field to help by holding additional infection control courses to continue and stabilize the infection control training program. Using the educational model is a good guide for developing and implementing and evaluating educational courses.

There are different educational models such as ADDIE, Kern, and ASSURE which cover all stages of the implementation and evaluation of the educational program [43]. Although some researchers stated that Kern's model is a general, short and practical approach [44, 45]. Finally, it is recommended that professors and clinical managers pay attention to the context, goals, approaches, evaluation and previous studies of the application of the educational model in order to choose the appropriate educational model [46].

## Limitations and strengths

The strengths of the present study are first, the development of the infection control program was based on the needs assessment of nursing students. Second, the use of Kern's model in the design, implementation and evaluation of the program, and third, measuring the outcomes in 2 weeks and 2 months later. Limitation of the study can be pointed out as a single group. One of our limitations was the small sample size in the quantitative phase. Also, the participating nursing students in the qualitative phase were from a midwifery nursing school in the west of Iran, so it cannot be generalized to all societies. In addition, we used the evaluation method of checking students' assignments and conducting pre-test and post-test in this study. It is recommended to use evaluation methods such as 360 degrees in future studies. We evaluated the infection control program 2 weeks and 2 months after the intervention. Future experimental studies should conduct the evaluation for a longer period of time, for example, 6 months of follow-up.

## Conclusion

According to our results and the fact that various studies have recommended the need to revise the curriculum for the integration of the infection control program in the undergraduate nursing education, it is suggested that the infection control program be included in the nursing program in the field of community health nursing. Of course, it is necessary to conduct more studies in the field by dividing this program into internship and field internship.

## Data Availability

Data were generated at Shahrekord University of Medical Sciences. Derived data supporting the findings of this study are available from the corresponding author [Haydeh Heidari] on request.
